# Early atherosclerosis in systemic sclerosis and its relation to disease or traditional risk factors

**DOI:** 10.1186/ar2408

**Published:** 2008-04-25

**Authors:** Martha E Hettema, Dan Zhang, Karina de Leeuw, Ymkje Stienstra, Andries J Smit, Cees GM Kallenberg, Hendrika Bootsma

**Affiliations:** 1 Department of Internal Medicine, Division of Rheumatology and Clinical Immunology, University Medical Center Groningen, University of Groningen, Hanzeplein 1, PO Box 30.001, 9700 RB Groningen, The Netherlands; 2 Department of Internal Medicine, University Medical Center Groningen, University of Groningen, Hanzeplein 1, PO Box 30.001, 9700 RB Groningen, The Netherlands; 3 Department of Internal Medicine, Division of Vascular Diseases, University Medical Center Groningen, University of Groningen, Hanzeplein 1, PO Box 30.001, 9700 RB Groningen, The Netherlands

## Abstract

**Introduction:**

Several systemic autoimmune diseases are associated with an increased prevalence of atherosclerosis which could not be explained by traditional risk factors alone. In systemic sclerosis (SSc), microvascular abnormalities are well recognized. Previous studies have suggested an increased prevalence of macrovascular disease as well. We compared patients with SSc to healthy controls for signs of early atherosclerosis by measuring intima-media thickness (IMT) of the common carotid artery in relation to traditional risk factors and markers of endothelial activation.

**Methods:**

Forty-nine patients with SSc, of whom 92% had limited cutaneous SSc, and 32 healthy controls were studied. Common carotid IMT was measured by using B-mode ultrasound. Traditional risk factors for cardiovascular disease were assessed and serum markers for endothelial activation were measured.

**Results:**

In patients with SSc, the mean IMT (median 0.69 mm, interquartile range [IQR] 0.62 to 0.79 mm) was not significantly increased compared with healthy controls (0.68 mm, IQR 0.56 to 0.75 mm; *P *= 0.067). Also, after correction for the confounders age, high-density lipoprotein (HDL) cholesterol, and low-density lipoprotein cholesterol (*P *= 0.328) or using a different model taking into account the confounders age, HDL cholesterol, and history of macrovascular disease (*P *= 0.474), no difference in IMT was present between SSc patients and healthy controls. Plaques were found in three patients and not in healthy controls (*P *= 0.274). In patients, no correlations were found between maximum IMT, disease-related variables, and markers of endothelial activation. Endothelial activation markers were not increased in SSc patients compared with controls.

**Conclusion:**

SSc is not associated with an increased prevalence of early signs of atherosclerosis.

## Introduction

Systemic sclerosis (SSc) is a generalized connective tissue disorder characterized by fibrosis of the skin and internal organs and widespread vascular lesions. The pathogenesis of the vasculopathy is not fully understood, but a viral trigger, immune reactions to viral or environmental factors, reperfusion injury, or antiendothelial antibodies may all be involved [1]. Also, angiogenesis is insufficient or defective [2,3]. Most attention has been given to microvascular disease in SSc, but previous studies have suggested an increased prevalence of macrovascular disease as well [4,5].

In other autoimmune diseases, such as systemic lupus erythematosus (SLE), rheumatoid arthritis, and Wegener granulomatosis, a significantly increased prevalence of atherosclerosis has been described [6-10]. Atherosclerosis nowadays is considered an inflammatory disease in which endothelial cell dysfunction is strongly implicated in its pathogenesis [11], in part related to traditional risk factors like smoking and dyslipidemia. Because of the increased cardiovascular morbidity and mortality in the aforementioned autoimmune diseases, attention has been given to the presence and treatment of cardiovascular risk factors. Despite increased mortality rates in SSc (partly due to cardiac involvement), cardiovascular risk factors and the presence of macrovascular disease have been emphasized less [12,13]. In this study, we assessed signs of early atherosclerosis by measuring intima-media thickness (IMT) of the common carotid artery (CCA) in patients with SSc and healthy controls. In addition, we related the outcome to traditional risk factors and markers of endothelial activation.

## Materials and methods

### Patients

Consecutive patients with SSc, according to the American College of Rheumatology criteria [14], attending our outpatient clinic were included. Patients were subclassified in subsets as defined by LeRoy and colleagues [15,16]. Forty-nine patients were included. Pregnancy was an exclusion criterion. Healthy subjects were included in this study as controls. Ethical approval for the study was obtained from the Medical Ethical Committee of the University Medical Center Groningen (University of Groningen, Groningen, The Netherlands). Informed consent was obtained from each participant.

Data were obtained from all subjects with respect to traditional risk factors for cardiovascular disease (CVD), including body mass index (BMI), smoking status, diabetes, blood pressure, lipid levels, and family history of CVD (considered positive if first-degree relatives suffered from CVD before 60 years of age).

In patients with SSc, we assessed disease-related factors as possible determinants of macrovascular disease. To assess disease activity, the preliminary European Scleroderma Study Group (EScSG) activity index (a score ranging from 0 to 10) was used. A score higher than 3 denotes active disease [17,18]. Also, the revised preliminary SSc severity scale (Medsger severity scale), a measure of activity, damage, and severity, was used. This scale is a nine-organ disease severity scale in which for each organ system a score of 0 to 4 is applied, with 0 being normal and 4 denoting end-stage organ involvement [19]. We also recorded statin use, cumulative prednisolone dose, and current or former use of immunosuppressive agents.

### Measurements of intima-media thickness

B-mode ultrasonography was used to measure the common carotid IMT. Measurements were limited to the CCA and not extended to other segments. The prevalence of increased IMT and/or plaques is substantially higher in the carotid bulb or internal carotid artery (ICA), but the intended quantitative comparison between SSc patients and controls may be hindered by including these segments. An Acuson 128XP ultrasound device with a 7 MHz linear array transducer (Acuson Corporation, now part of Siemens Medical Solutions USA, Inc., Malvern, PA, USA) was used for measuring IMT in all patients and controls, as described before [7,20]. With the subjects in the supine position, the left CCA wall segment was scanned from a lateral transducer position and recorded on s-VHS tape. The CCA wall segment was defined as 1 cm proximal to the carotid bifurcation. The far wall of the left common artery was assessed at three different positions. Off-line video analysis, using a previously described image analysis program [21], was performed by one reader unaware of patient or control group data or characteristics. The highest IMT value found in this segment was considered to be the maximum IMT, and the mean of three measurements in this segment was considered to be the mean IMT.

### Blood analysis

Lipid levels were measured by routine techniques. Additional serum and plasma samples for determination of markers of endothelial activation were stored at -20°C until analysis. Serum levels of vascular cell adhesion molecule-1 (s-VCAM-1) (R&D Systems Europe Ltd, Abingdon, UK) and thrombomodulin (TM) (Diaclone, Besançon, France) were measured according to the manufacturers' instructions. Serum was used to determine C-reactive protein (CRP) and plasma was used to determine von Willebrand factor (vWF) using in-house enzyme-linked immunosorbent assays, as described before [7].

### Statistical analysis

Values are expressed as mean ± standard deviation when variables were normally distributed and as median with interquartile range (IQR) (25th to 75th percentile) in case of a non-normal distribution. Because the number of patients with diffuse cutaneous SSc (dSSc) was low in our patient group, subset analysis could not be performed. Differences between patients and controls were assessed by Student *t *test, Mann-Whitney *U *test, and χ^2 ^test (Pearson chi-square or Fisher exact test) as appropriate. Linear regression was used to assess the relationship between IMT and the different groups (SSc patients and healthy controls). An unadjusted analysis in which no corrections were made for possible confounders and an adjusted analysis in which corrections were made for possible confounders are presented. The variables were entered manually. The level of significance for the association between group and outcome variable was set at a *P *value of less than 0.05. The variables age, gender, BMI, and smoking were also studied as potential effect modifiers in the relationship of interest. The correlation between maximum IMT and disease-related factors and endothelial markers was assessed by Spearman rank correlation coefficient since maximum IMT was non-normally distributed. All analyses were carried out with the Statistical Package of Social Science, version 12.1. for Windows (SPSS Inc., Chicago, IL, USA).

## Results

### Characteristics of patients and controls

The demographic characteristics and the traditional risk factors of SSc patients and healthy controls are shown in Table 1. Patients tended to be older (55.4 ± 11.6 versus 50.9 ± 10.1 years; *P *= 0.078) and used significantly more statins (14% versus 0%; *P *= 0.038) than healthy controls. Patients had lower levels of high-density lipoprotein (HDL) cholesterol (1.40 mmol/L, IQR 1.23 to 1.80 mmol/L, versus 1.68 mmol/L, IQR 1.48 to 1.89 mmol/L; *P *= 0.027) and higher levels of triglycerides (1.36 mmol/L, IQR 1.16 to 2.14 mmol/L, versus 1.17 mmol/L, IQR 0.77 to 1.67 mmol/L; *P *= 0.030) than controls. No significant differences were found in other cardiovascular risk factors. Four patients had a history of macrovascular events compared with none in the control group.

**Table 1:** Traditional risk factors in systemic sclerosis patients and healthy controls

Characteristic	Patients (n = 49)	Controls (n = 32)	*P *value
Age, years	55.4 ± 11.6	50.9 ± 10.1	0.078
Male gender, number (percentage)	8 (16%)	3 (9.4%)	0.513
Body mass index, kg/m^2^	23.7 ± 2.9	24.0 ± 2.9	0.582
Smoking			
Pack years for smokers, number (percentage)	3 (6%)	2 (6.5%)	1.000
Median (range)	20 (1–48)	10 (6–14)	
Diabetes mellitus, number (percentage)	2 (4%)	0	0.523
Hypertension treated with antihypertensive agents, number (percentage)	12 (24%)	2 (6%)	0.120
Blood pressure, mm Hg			
Systolic	120 (110–135)	120 (110–125)	0.409
Diastolic	75 (70–80)	75 (70–80)	0.379
Cholesterol, mmol/L			
Total	5.22 ± 1.00	5.53 ± 1.06	0.217
Low-density lipoprotein	3.02 ± 0.85	3.06 ± 1.08	0.871
High-density lipoprotein	1.40 (1.23–1.80)	1.68 (1.48–1.89)	0.027
Triglycerides, mmol/L	1.36 (1.16–2.14)	1.17 (0.77–1.67)	0.030
Lipid-lowering drugs, number (percentage)	7 (14%)	0	0.038
Simvastatin, number (mean dose in milligrams)	4 (20)	0	
Atorvastatin, number (mean dose in milligrams)	2 (25)	0	
Rosuvastatin, number (mean dose in milligrams)	1 (10)	0	
Family history of cardiovascular disease, number (percentage)	10 (20%)	5 (16%)	0.603
Cardiovascular history, number (percentage)	4 (8%)	0	0.149
Cardiovascular	1		
Cerebrovascular	1		
Peripheral vascular disease	2		
C-reactive protein, mg/L	3.5 (1.6–7.0)	0.8 (0.3–2.0)	< 0.001

Unless stated otherwise, data are expressed as mean ± standard deviation when normally distributed and as median (interquartile range) when non-normally distributed.

Patients with SSc had a median disease duration of 6 years and had experienced Raynaud phenomenon for almost 11 years. Limited cutaneous SSc (lcSSc) was present in 92% of patients and diffuse cutaneous SSc was present in 8% of patients. Patients had a median modified Rodnan skin score of 7.0 (IQR 4.5 to 14.0), a preliminary EScSG disease activity index of 0.5 (IQR 0.5 to 1.75), and a revised preliminary SSc severity scale score (Medsger severity scale score) of 6.0 (IQR 4.5 to 7.0). The preliminary EScSG disease activity index may have been an underestimation of reality since not all variables (especially erythrocyte sedimentation rate [ESR] and complement) were available. As shown in Table 1, CRP levels were not substantially elevated, suggesting normal ESR levels. However, patients had significantly higher CRP levels than controls (3.5, IQR 1.6 to 7.0, versus 0.8, IQR 0.3 to 2.0; *P *< 0.001). Forty-three percent of patients were current or former users of prednisolone, with a median cumulative dose of 3.6 g (1.9 to 16.1 g) (Table 2).

**Table 2:** Characteristics of patients with systemic sclerosis

Disease characteristic	N = 49
Systemic sclerosis subset, number (percentage)	
Diffuse cutaneous systemic sclerosis	4 (8%)
Limited cutaneous systemic sclerosis	45 (92%)
Disease duration, years	6 (2–12)
Raynaud phenomenon duration, years	11 (6–25)
Antibody, number (percentage)	
Scl70 (topoisomerase 1)	4 (8%)
Centromere	22 (45%)
Nuclear ribonucleoprotein	2 (4%)
Antinuclear antibodies, not specified	17 (35%)
None	4 (8%)
EScSG disease activity index	0.5 (0.5–1.5)
Medsger severity scale score	6.0 (4.5–7.0)
Modified Rodnan skin score	7.0 (4.5–14.0)
Prednisolone use, number (percentage)	
None	28 (57%)
Former	13 (27%)
Current	8 (16%)
Cumulative prednisolone dose, grams	3.6 (1.9–16.1)
Immunosuppressive agents, number (percentage)	
Never used	25 (51%)
Former or current users	24 (49%)
Methotrexate	
Current	12
Former	7
Cyclophosphamide	
Current	2
Former	4
Azathioprine	
Current	3
Former	3
Cyclosporin	
Current	0
Former	1

Unless stated otherwise, data are expressed as mean ± standard deviation when normally distributed and as median (interquartile range) when non-normally distributed. EScSG, European Scleroderma Study Group.

### Intima-media thickness

**Figure 1:**
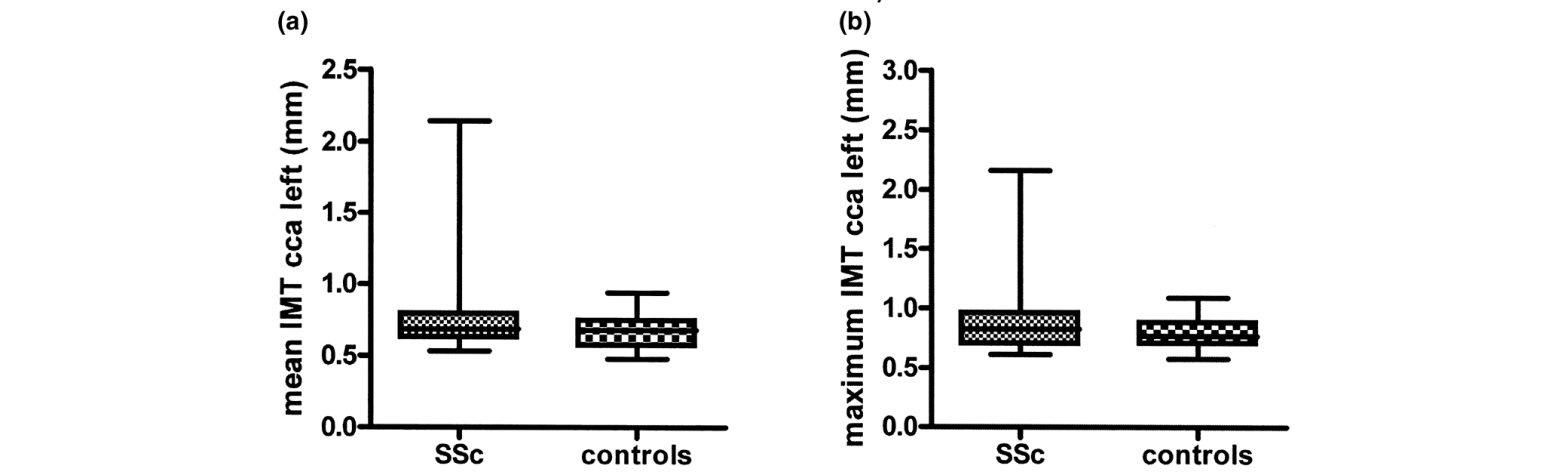
Box plots of **(a) **mean and **(b) **maximum left common carotid artery (CCA) intima-media thickness (IMT) of systemic sclerosis (SSc) patients and healthy controls. Data are uncorrected for confounders. The median, interquartile range, and minimum and maximum values are shown.

The median values for the mean IMT measurements in the CCA were 0.69 mm (IQR 0.62 to 0.79 mm) in patients and 0.68 mm (IQR 0.56 to 0.75 mm) in controls (Figure 1a). Also, IMT values were comparable between SSc patients and controls for each age decade. Among the four outliers in the group of SSc patients with an IMT of greater than 1.10 mm, one patient had a history of a cerebrovascular accident and one patient was known to have left ventricular dysfunction probably caused by coronary artery disease. Both had other cardiovascular risk factors like current or former smoking and hypertension. The other two patients were not known to have clinically manifest CVDs. One of these patients had a family history of CVD. Other risk factors were not present. Although plaques were not a primary endpoint, they were observed in three patients and not in healthy controls. Linear regression analysis of the mean IMT in the CCA between controls and patients, when not corrected for possible confounders, demonstrated no significant difference in mean IMT (B = 0.101; *P *= 0.067) (Table 3). Also, no significant differences were seen between the groups after correction for the strongest confounders. Both in the model with the confounders age, HDL cholesterol, and low-density lipoprotein (LDL) cholesterol (B = 0.042; *P *= 0.328) and in the model with the confounders age, HDL cholesterol, and history of macrovascular disease (B = 0.030; *P *= 0.474), no differences were found between patients and healthy controls. The possible relationship between mean IMT and SSc patients versus controls was lost when traditional risk factors were entered into the regression analysis. The addition of the confounder history of hypertension did not change the outcome. No correction for systolic and diastolic blood pressure, use of statins, smoking, diabetes mellitus, gender, BMI, total cholesterol, or triglycerides was necessary since these outcome parameters were not confounders in the linear regression model. No effect modification by age, gender, BMI, or smoking was found.

**Table 3:** Linear regression analysis for risk factors for mean intima-media thickness of the left common carotid artery in systemic sclerosis patients and healthy controls

Group (controls, patients)	B^a^	95% confidence interval	*P *value
Unadjusted/crude	0.101	-0.007, 0.209	0.067
Adjusted^b^	0.042	-0.043, 0.128	0.328
Adjusted^c^	0.030	-0.054, 0.114	0.474

^a^B is the regression coefficient. In the adjusted models, corrections were made for the confounders ^b^age, high-density lipoprotein (HDL) cholesterol, and low-density lipoprotein cholesterol or ^c^age, HDL cholesterol, and history of macrovascular disease; a total of 66 patients and controls were available for this analysis.

No significant differences were seen for the maximum IMT of the CCA between SSc patients (0.83 mm, IQR 0.70 to 0.97 mm) and healthy controls (0.77 mm, IQR 0.70 to 0.88 mm) by means of linear regression analysis before or after correction for the confounders age, HDL cholesterol, gender, and history of macrovascular disease (Figure 1b and Table 4). The addition of the other confounders (that is, history of hypertension, diastolic blood pressure, and LDL cholesterol) did not change the outcome. In patients, no correlations were found between maximum IMT, CRP, and disease-related variables.

**Table 4:** Linear regression analysis unadjusted and adjusted for risk factors for maximum intima-media thickness of the left common carotid artery in systemic sclerosis patients and healthy controls

Group (controls, patients)	B^a^	95% confidence interval	*P *value
Unadjusted/crude	0.101	-0.017, 0.219	0.093
Adjusted^b^	0.028	-0.071, 0.127	0.577
Adjusted^c^	0.022	-0.081, 0.126	0.668

^a^B is the regression coefficient. In the adjusted models, corrections were made for the confounders ^b^age, high-density lipoprotein (HDL) cholesterol, and gender or ^c^age, HDL cholesterol, and history of macrovascular disease; a total of 66 patients and controls were available for this analysis.

### Endothelial activation markers

**Figure 2:**
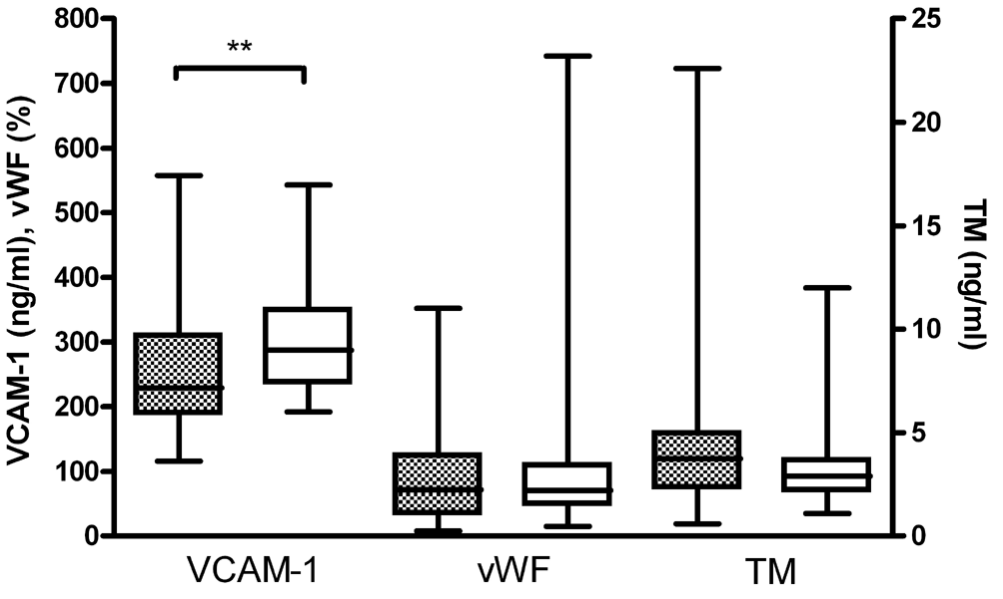
Endothelial activation markers. Boxes indicate the median value and the interquartile ranges. Lines indicate the minimum and maximum values. Dotted bars represent systemic sclerosis patients and open bars represent controls. TM, thrombomodulin; VCAM-1, vascular cell adhesion molecule-1; vWF, von Willebrand factor. **P < 0.05.

Markers of endothelial activation were not increased in patients with SSc. Compared with controls, levels of VCAM-1 were even decreased (229 ng/mL, IQR 188 to 311 ng/mL, versus 287 ng/mL, IQR 236 to 350 ng/mL; *P *= 0.014). No differences between SSc patients and controls were found in levels of vWF (72%, IQR 34% to 125%, versus 71%, IQR 48% to 110%; *P *= 0.691) and TM (3.8 ng/mL, IQR 2.3 to 5.0 ng/mL, versus 2.9 ng/mL, IQR 2.1 to 3.7 ng/mL; *P *= 0.151) (Figure 2). Levels of endothelial markers were not correlated with maximum IMT.

## Discussion

This study did not show differences in the IMT of the CCA and prevalence of plaques between patients with SSc and healthy controls, suggesting no increased prevalence of early atherosclerotic macrovascular disease in SSc. Also, no correlations were found between IMT, disease-related factors, and markers of endothelial activation. Traditional risk factors, like increasing age and dyslipidemia, accounted for increased IMT values in SSc patients and controls.

After the first reports suggesting an increased prevalence of macrovascular involvement in SSc, several studies have been performed in the last decade using IMT of the carotid artery as a marker of early atherosclerosis. Lekakis and colleagues [22], Kaloudi and colleagues [23], and Bartoli and colleagues [24,25] found strongly increased IMT values in the CCA in SSc patients compared with controls. In these studies, mean IMT values were markedly higher than in our patients whereas mean age was comparable. It is not known whether patient groups in studies by Kaloudi and colleagues [23] and Bartoli and colleagues [24,25] are from overlapping cohorts since these studies were performed in the same center and published in the same period. A larger percentage of diffuse cutaneous systemic sclerosis (dcSSc) subtype was present in these studies compared with our study, although Kaloudi and colleagues [23] found no significant differences between mean IMT between subtypes. On the other hand, our results are in agreement with those of Cheng and colleagues [26,27] and Szucs and colleagues [28], who found no differences in IMT values in SSc patients compared with controls. Apart from a younger age and a larger percentage of dcSSc subtype in the study by Cheng and colleagues [27], age was comparable, as were IMT values. No difference was present in IMT values between subsets in this study either [26]. In view of this findings and given the small number of patients with dcSSc in our study, we did not perform a subset analysis. Overall, these discrepancies between studies in the presence of early atherosclerosis as measured by common carotid IMT in SSc patients might be explained by methodological differences, such as patients included in the study and comorbidity.

In our SSc patients, lipid levels and statin use were statistically different from healthy controls. After correction for the strongest confounders in our model, no differences were seen in IMT values between SSc patients and healthy controls. Although statin use was no confounder in our model, a meta-analysis showed that statin therapy is efficient in decreasing the rate of carotid atherosclerosis progression in the long term [29]. Otherwise, statins may have a potential benefit in preventing endothelial dysfunction in SSc patients [30].

Treatment with immunosuppressive agents, especially corticosteroids, influences the atherogenic process. Corticosteroids are considered to have atherogenic properties [31], like azathioprine [32], whereas for hydroxychloroquine [31] and methotrexate [9], a protective effect against atherosclerosis has been described. Otherwise, immunosuppressive therapy with prednisolone, cyclophosphamide, or hydroxychloquine was associated with the absence of plaques in patients with SLE [33]. It is difficult to establish whether the observed associations between immunosuppressive agents and atherosclerosis are due to the immunosuppressive agents themselves or to their effect on the activity of the autoimmune disease. In our study, 49% were former or current users of immunosuppressive agents. No association was found between maximum IMT and cumulative prednisolone dose and use of other immunosuppressive agents.

Markers of inflammation, such as CRP, are related to the risk of cardiovascular and peripheral vascular disease. Increased levels of CRP are associated with increased risk of symptomatic disease [34,35]. In our population, CRP levels were significantly elevated compared with healthy controls. The CRP levels we found might have been associated with future coronary events [34,35], but we found no association between CRP and IMT values. This can be explained by the study design. Our study was not designed to find a relationship between CRP and risk of CVD, and we did not exclude other conditions that could explain elevated CRP levels, like intercurrent infections. Otherwise, in SSc patients, besides elevations due to infection, no significant elevations of CRP levels are seen [36].

Surprisingly, we did not find elevated levels of endothelial activation markers. Our population of SSc patients was heterogeneous with respect to disease duration. The typical patient had inactive disease. Most inflammation is expected in the early stages of the disease or in patients with active disease. Also, by using the Medsger severity scale, we could not find an association between macrovascular disease and the severity of SSc. This might explain the absence of increased levels of endothelial activation markers. All these data point to the absence of premature atherosclerosis in SSc.

Our results might be an underestimation of atherosclerosis in SSc patients and controls. Besides the possible explanations as stated above, our patients were suffering predominantly from lcSSc, in which inflammation is not always present [37]. However, when IMT values were analyzed in subsets, other authors did not find differences in these values between subsets [23,26]. Furthermore, we used IMT values of the CCA. This segment is commonly evaluated in our laboratory as it can be approached easily and measurements on this segment are reproducible. Using the same protocol as described here, we found increased IMT values in patients with SLE [38]. However, atherosclerotic lesions appear later in the CCA than in the ICA or bulb, but these latter two segments are more difficult to visualize [39]. Also, it can be difficult to assess whether IMT of the CCA represents atherosclerosis or vascular hypertrophy [40]. Although other noninvasive markers of early changes in the arterial wall are available (such as arterial wall thickening and stiffening), carotid IMT has been used more frequently and has been found to be a strong predictor of future vascular events [40,41].

## Conclusion

IMT of the CCA is not increased in patients with SSc compared with controls, either when uncorrected or corrected for traditional risk factors. So, SSc seems not to be associated with increased early atherosclerotic macrovascular disease. It seems that, although SSc is characterized by endothelial dysfunction, this is reflected mainly in microvascular disease.

## Abbreviations

BMI = body mass index; CCA = common carotid artery; CRP = C-reactive protein; CVD = cardiovascular disease; dcSSc = diffuse cutaneous systemic sclerosis; EScSG = European Scleroderma Study Group; ESR = erythrocyte sedimentation rate; HDL = high-density lipoprotein; ICA = internal carotid artery; IMT = intima-media thickness; IQR = interquartile range; lcSSc = limited cutaneous systemic sclerosis; LDL = low-density lipoprotein; SLE = systemic lupus erythematosus; SSc = systemic sclerosis; TM = thrombomodulin; VCAM-1 = vascular cell adhesion molecule-1; vWF = von Willebrand factor.

## Competing interests

The authors declare that they have no competing interests.

## Authors' contributions

MEH participated in the conception and design of the study, participated in the recruitment of patients and controls and data collection, helped to conduct the statistical analysis, and was involved in drafting the manuscript or revising it critically. KdL participated in the conception and design of the study, participated in the recruitment of patients and controls and data collection, helped to perform enzyme-linked immunosorbent assay (ELISA) experiments, and was involved in drafting the manuscript or revising it critically. AJS, CGMK, and HB participated in the conception and design of the study and were involved in drafting the manuscript or revising it critically. DZ participated in the recruitment of patients and controls and data collection and helped to perform ELISA experiments. YS helped to conduct the statistical analysis. All authors read and approved the final manuscript.
